# Exposure to violence and risk of post-traumatic stress disorder in family caregivers of psychotic patients

**DOI:** 10.1192/j.eurpsy.2021.1203

**Published:** 2021-08-13

**Authors:** M. Daoud, N. Charfi, S. Omri, R. Feki, N. Smaoui, M. Maalej Bouali, L. Zouari, J. Ben Thabet, M. Maalej

**Affiliations:** 1 Department Of Psychiatry “c”, Universty Hospital of Hedi Chaker, sfax, Tunisia; 2 Psychiatry C Department, Hedi chaker University hospital, sfax, Tunisia

**Keywords:** Aggression, caregivers, psychosis, post-traumatic stress disorder

## Abstract

**Introduction:**

Family caregivers of psychotic patients are exposed to violence and stress. However, associated psychological outcomes are poorly characterized in this population.

**Objectives:**

The aim of this study was to clarify the relationship between violence directed towards caregivers of patients with psychosis and developing post-traumatic stress disorder (PTSD).

**Methods:**

Participants were family caregivers of psychotic patients (n=95). They completed a questionnaire assessing sociodemographic characteristics. Sociodemographic and clinical data of patients were collected from medical records. We used the perceptions of prevalence of aggression scale (POPAS) to measure the frequency and severity of aggression directed at the respondent in the past and the Impact of Event Scale-Revised (IES-R) to evaluate PTSD.

**Results:**

A rate of 75.8% of caregivers reported experiencing moderate to severe levels of aggression. Decreased contact with patient (p=0.00), male gender (p=0.00), older age (p=0.00) and parent relationship (p=0.01) of caregivers, diagnosis of schizophrenia or schizoaffective disorder (p=0,00) and poor adherence to treatment (p=0,00) in affected relatives were associated with experiences of moderate–severe aggression. More than a half of caregivers (54.7%) reported potentially significant levels of PTSD which correlated with the level of aggression (p=0.00).

**Conclusions:**

Our findings suggest that a large proportion of family caregivers of patient-initiated violence in psychosis reported experiencing a great distress and a high level of PTSD symptomatology. So, more attention should be paid to the support needs of caregivers who are faced with potentially life threatening aggressive behaviour by psychotic family members.

